# The impact of the COVID-19 pandemic on antimicrobial resistance: a debate

**DOI:** 10.1093/jacamr/dlaa053

**Published:** 2020-07-17

**Authors:** David van Duin, Gavin Barlow, Dilip Nathwani

**Affiliations:** d1 Division of Infectious Diseases, University of North Carolina, Chapel Hill, NC, USA; d2 Department of Infection, Hull University Teaching Hospitals NHS Trust, Hull, UK and Hull York Medical School, University of York, Heslington, UK; d3 Emeritus Honorary Professor of Infection, University of Dundee, Ninewells Hospital and Medical School, Dundee, UK

## Abstract

The coronavirus disease 2019 (COVID-19) pandemic is having an enormous impact on public health. Infection with SARS-CoV-2 has become a leading cause of morbidity and mortality in many regions around the world. As many COVID-19 patients are treated with antibiotics, there is concern regarding an associated rise in rates of antimicrobial resistance (AMR). On the other hand, social distancing, isolation and reduced travel may result in decreased spread of AMR. In this issue of *JAC-Antimicrobial Resistance*, we present a PRO/CON debate on the question of the potential impact of COVID-19 on AMR rates.

In December 2019, a cluster of patients infected with a novel coronavirus was recognized in Wuhan, China.^1^ This novel coronavirus was found to be similar to the Severe Acute Respiratory Syndrome (SARS)-coronavirus (CoV) and was named SARS-CoV-2. The associated disease was named coronavirus disease 2019 (COVID-19). COVID-19 spread rapidly worldwide and at the time of writing over 6 000 000 cases have been confirmed, with over 300 000 deaths.^2^ The impact of the COVID-19 pandemic on antimicrobial resistance (AMR) remains unclear.

Antibiotic use, often to treat presumed secondary bacterial coinfection, is common in patients infected with SARS-CoV-2. This is despite the relatively low rates of these infections. Furthermore, early reports of the possible activity of hydroxychloroquine in COVID-19 when used in combination with azithromycin led to substantial use of the latter.^3^ Another important feature of this disease is the prolonged dependence on invasive mechanical ventilation of patients with severe infection. In a large cohort, 12% of hospitalized patients in New York State required mechanical ventilation.^4^ In a smaller Boston cohort of COVID-19 patients who were managed with invasive mechanical ventilation, the median duration was 16 days (IQR: 10.0–21.0 days).^5^ These prolonged intensive care stays, high mortality rate, diagnostic and prognostic uncertainty and concern for secondary bacterial infections has led to frequent empiric antibacterial use. In Chinese randomized trials evaluating remdesivir and lopinavir/ritonavir, over 90% of patients were treated with antibacterials.^6^^,^^7^ In an analysis of published literature, Rawson *et al.*^8^ report that while only 8% of patients included in publications had reported bacterial or fungal coinfection, 72% of patients were treated with antibiotics. This frequent use of empiric antibiotics, often broad spectrum or in combination, has led to concerns over increased AMR rates, with an attendant call not to neglect antimicrobial stewardship, which perhaps now has an even more important role.^9^

On the other hand, the COVID-19 pandemic has resulted in unprecedented changes in society that may actually result in decreased AMR rates. Social distancing, a focus on isolation and reductions in national and international travel may decrease the spread of AMR pathogens and associated AMR genes. International travel has been consistently reported as an important risk factor for acquisition of AMR pathogens.^10^ In April 2020, the number of travellers going through US checkpoints was reduced by 95% compared with 2019.^11^ In addition, social distancing measures to limit the spread of SARS-CoV-2 has impacted prevalence of other infectious diseases, most notably influenza.^12^ These measures may similarly impact the spread of AMR.

In this issue of *JAC-Antimicrobial Resistance*, we have invited international thought leaders for a PRO/CON debate regarding the impact of COVID-19 on AMR. Clancy and colleagues^13^ argue in favour of the premise that COVID-19 will result in an overall increase in AMR rates. They argue that while overall hospital attendance may have decreased, the patients who are admitted during the pandemic have a higher likelihood of being treated with broad-spectrum antibiotics, as well as being exposed to procedures, such as invasive mechanical ventilation, that are known risk factors for AMR development. In addition, many shared risk factors exist for patients who are at risk of COVID-19 requiring hospitalization and those at risk of AMR. Collignon and Beggs^14^ argue that COVID-19 will not lead to increased AMR rates. They present several important arguments, ranging from factors that impact transmission in healthcare and community settings, to the impact of decreased international travel. They also emphasize that control of AMR requires control of the spread of AMR pathogens, in addition to antimicrobial stewardship.

The impact of COVID-19 on AMR rates remains to be determined and is likely to be heterogeneous due to variation in healthcare practices, such as in the specific antimicrobials used and infection prevention and control interventions during the pandemic. It is clearly an important area for future systematic studies.

## Transparency declarations

D.v.D. reports being on the advisory Board for Allergan, Achaogen, Qpex, Shionogi, Tetraphase, Sanofi-Pasteur, T2 Biosystems, NeuMedicine, Roche, MedImmune, Astellas and Merck. D.N. and G.B.: none to declare.

## References

[1] Huang C, Wang Y, Li X et al Clinical features of patients infected with 2019 novel coronavirus in Wuhan, China. Lancet 2020; 395: 497–506. 31986264 10.1016/S0140-6736(20)30183-5 PMC7159299

[2] Johns Hopkins University. Coronavirus Resource Center. https://coronavirus.jhu.edu/.

[3] Rodrigo C, Fernando SD, Rajapakse S. Clinical evidence for repurposing chloroquine and hydroxychloroquine as antiviral agents: a systematic review. Clin Microbiol Infect 2020; doi: 10.1016/j.cmi.2020.05.016. 10.1016/j.cmi.2020.05.016 PMC7250111 32470568

[4] Richardson S, Hirsch JS, Narasimhan M et al Presenting characteristics, comorbidities, and outcomes among 5700 patients hospitalized with COVID-19 in the New York City area. JAMA 2020; doi: 10.1001/jama.2020.6775. 10.1001/jama.2020.6775 PMC7177629 32320003

[5] Ziehr DR, Alladina J, Petri CR et al Respiratory pathophysiology of mechanically ventilated patients with COVID-19: a cohort study. Am J Respir Crit Care Med 2020; doi: 10.1164/rccm.202004-1163LE. 10.1164/rccm.202004-1163LE PMC7301734 32348678

[6] Cao B, Wang Y, Wen D et al A trial of lopinavir-ritonavir in adults hospitalized with severe Covid-19. N Engl J Med 2020; 382: 1787–99. 32187464 10.1056/NEJMoa2001282 PMC7121492

[7] Wang Y, Zhang D, Du G et al Remdesivir in adults with severe COVID-19: a randomised, double-blind, placebo-controlled, multicentre trial. Lancet 2020; 395: 1569–78. 32423584 10.1016/S0140-6736(20)31022-9 PMC7190303

[8] Rawson TM, Moore LSP, Zhu N et al Bacterial and fungal co-infection in individuals with coronavirus: a rapid review to support COVID-19 antimicrobial prescribing. Clin Infect Dis 2020; doi: 10.1093/cid/ciaa530. 10.1093/cid/ciaa530 PMC7197596 32358954

[9] Huttner BD, Catho G, Pano-Pardo JR et al COVID-19: don’t neglect antimicrobial stewardship principles! Clin Microbiol Infect 2020; doi: 10.1016/j.cmi.2020.04.024. 10.1016/j.cmi.2020.04.024 PMC7190532 32360446

[10] Frost I, Van Boeckel TP, Pires J et al Global geographic trends in antimicrobial resistance: the role of international travel. J Travel Med 2019; 26: taz036. 31115466 10.1093/jtm/taz036

[11] Airlines for America. Impact of COVID-19. https://www.airlines.org/dataset/impact-of-covid19-data-updates/#.

[12] Noh JY, Seong H, Yoon JG et al Social distancing against COVID-19: implication for the control of influenza. J Korean Med Sci 2020; 35: e182. 32419400 10.3346/jkms.2020.35.e182 PMC7234863

[13] Clancy CJ, Buehrle DJ, Nguyen MH. PRO: the COVID-19 pandemic will result in increased antimicrobial resistance rates. JAC Antimicrob Resist 2020; doi: 10.1093/jacamr/dlaa049. 10.1093/jacamr/dlaa049 PMC7454644 34192248

[14] Collignon P, Beggs JJ. CON: COVID-19 will NOT result in increased AMR prevalence. JAC Antimicrob Resist 2020; doi: 10.1093/jacamr/dlaa051. 10.1093/jacamr/dlaa051 PMC7454599 34192249

